# Papillary craniopharyngioma in a 4-year-old girl with BRAF V600E mutation: a case report and review of the literature

**DOI:** 10.1007/s00381-018-3925-4

**Published:** 2018-08-01

**Authors:** R. Borrill, E. Cheesman, S. Stivaros, I. D. Kamaly-Asl, K. Gnanalingham, John-Paul Kilday

**Affiliations:** 1 Department of Haematology/Oncology, Royal Manchester Children’s Hospital, Oxford Road, Manchester, England M13 9WL UK; 2 Children’s Brain Tumour Research Network (CBTRN), Royal Manchester Children’s Hospital, Oxford Road, Manchester, England UK; 3 The Centre for Paediatric, Teenage and Young Adult Cancer, Division of Cancer Sciences, The University of Manchester, Manchester, England UK; 4 Department of Histopathology, Royal Manchester Children’s Hospital, Oxford Road, Manchester, England M13 9WL UK; 5 Division of Informatics, Imaging and Data Sciences, School of Health Sciences, Faculty of Biology, Medicine and Health, University of Manchester, Manchester Academic Health Science Centre, Manchester, UK; 6 Academic Unit of Paediatric Radiology, Royal Manchester Children’s Hospital, Manchester University Hospitals NHS Foundation Trust, Manchester Academic Health Sciences Centre, Manchester, UK; 7 Department of Neurosurgery, Royal Manchester Children’s Hospital, Oxford Road, Manchester, England M13 9WL UK; 8 Department of Neurosurgery, Salford Royal Hospital, Stott Lane, Salford, England M6 8HD UK

**Keywords:** Papillary, Craniopharyngioma, Pediatric, BRAF V600E mutation

## Abstract

**Introduction:**

Craniopharyngiomas are one of the most frequently diagnosed hypothalamo-pituitary tumors in childhood. The adamantinomatous histological subtype accounts for most pediatric cases, while the papillary variant is almost exclusively diagnosed in adults. Here, we report a case of papillary craniopharyngioma in a very young child, confirmed by molecular tissue analysis.

**Case report:**

A 4-year-old girl was being investigated for symptomatic central hypothyroidism. Brain MR imaging revealed a large solid/cystic suprasellar mass, splaying the optic chiasm and measuring 3 × 1.9 × 2.3 cm. The patient underwent a transsphenoidal near total resection of the lesion, which was encased within a tumor capsule. Post-operatively, the patient developed transient diabetes insipidus but otherwise recovered well. The pathology of the lesion was consistent with a papillary craniopharyngioma with regions of stratified squamous epithelium accompanied by superficial goblet cells and ciliated cells. Subsequent next-generation sequencing analysis of the lesion confirmed the presence of a BRAF V600E mutation (BRAFc.1799T>A p. (Val600Glu). To date, she remains free from progression 1 year following surgery.

**Conclusion:**

This is the youngest case published to date of papillary craniopharyngioma with a confirmed BRAF V600E mutation. The case encourages discussion about the most appropriate adjuvant therapy for tumor progression in such cases, given the risks of radiotherapy to the developing brain and the increasing availability of oral BRAF inhibitor therapy.

## Introduction

Craniopharyngiomas are frequently diagnosed hypothalamo-pituitary tumors of childhood [12]. Despite a benign histology, their management presents an ongoing challenge given lesional proximity to critical structures. Disease progression or surgical intervention can result in neuro-endocrinological symptoms, visual disturbances, and obstructive hydrocephalus, which impacts on quality of life [9, 13, 22]. The apparent futility of chemotherapy [10], and concerns regarding adverse sequelae from adjuvant radiotherapy, particularly for the developing brain, compound the dilemma [14, 20].

Two histopathological variants are recognized [17]. Adamantinomatous craniopharyngiomas account for most pediatric cases [23]. Lesions often consist of cystic elements and are characterized by distinctive epithelial appearances featuring dense whorls merged with looser stellate reticulum, cholesterol crystals, and wet keratin [17]. In contrast, squamous papillary craniopharyngiomas are almost exclusive to adults. They are typically solid and homogenous in composition, comprising well-differentiated squamous epithelium without calcification, whorls, or wet keratin. Biological distinctions are also evident; WNT pathway aberrations are associated with adamantinomatous craniopharyngiomas [11], while up to 95% of papillary lesions harbor BRAF V600E mutations [6].

Here, we report one of the youngest patients to date with a papillary craniopharyngioma, confirmed by histological and molecular analysis. We demonstrate its rarity by literature review and discuss adjuvant therapeutic implications for affected young children, given the concerns regarding conventional radiotherapy and the advent of novel targeted therapy.

## Case report

A 4-year-old girl presented with symptomatic central hypothyroidism and short stature. Initial examination was unremarkable, including normal visual field assessment on confrontation testing.

**Fig. 1:**
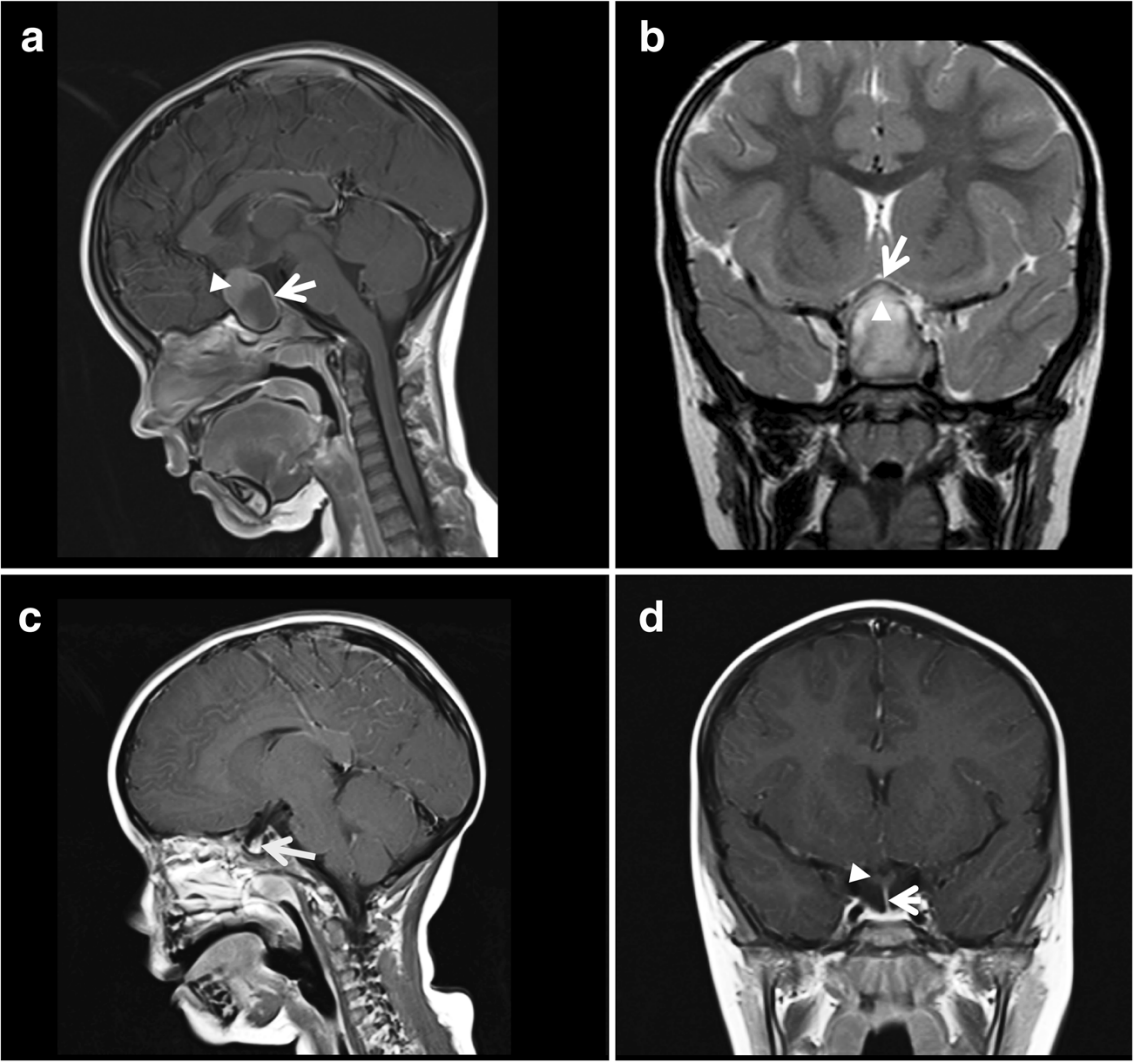
Sagittal pre-operative post-contrast T1 MRI brain scan image at presentation (**a**) revealing a large suprasellar lesion, measuring 3 × 1.9 × 2.3 cm. The lesion compromised a superior, solid component which had increased T1 signal pre-contrast (solid arrow head). This component also demonstrated high T2/FLAIR signal. An inferior, low FLAIR signal cystic component was noted. Peripheral rim enhancement of the lesion can be appreciated (white open arrow). **b** is a coronal T2 image also obtained at presentation which shows the upwards extend of the lesion (solid arrow) impinging onto and splaying the optic chiasm (open white arrow). Following transsphenoidal tumor surgery, follow-up sagittal T1 post-contrast MRI imaging (**c**) demonstrates improved appearances, with a small degree of non-enhancing residual tissue extending down into an enlarged pituitary sella (white arrow). The coronal T1 post-contrast image (**d**) now demonstrates a normal position of the optic chiasm (solid arrow) and a clearly discernable pituitary stalk which is slightly deviated to the left (white open arrow)

Assessment of pituitary functioning confirmed central hypothyroidism, but also identified cortisol and growth hormone deficiency. Subsequent gadolinium-enhanced magnetic resonance imaging (MRI) of the brain identified a solid/cystic suprasellar lesion, measuring 3 × 1.9 × 2.3 cm (Fig. 1a). The superior, solid aspect demonstrated increased T1 signal, while high FLAIR signal and peripheral contrast enhancement were noted in the more cystic, inferior component. The optic chiasm was splayed over the lesion. The ventricular system was normal.

The patient underwent a transsphenoidal near total resection of the mass, which was identified within a thick capsule, atypical for an adamantinomatous craniopharyngioma. Opening the lesion revealed a necrotic, cream-like material which was removed internally by suction and curettage. The residual cyst wall was unable to be completely dissected from surrounding structures.

The patient recovered well post-operatively, developing only transient diabetes insipidus which resolved within days. She had no neurological deficits following resection and post-operative visual field testing was comparable to presentation. The patient subsequently commenced levothyroxine and hydrocortisone therapy, while growth hormone replacement was planned to commence once clinical and radiological stability were confirmed.

Post-operative MRI scans showed residual enhancing cyst wall but no measurable solid component (Fig. 1b). To date, the patient remains clinically and radiologically stable with no evidence of disease progression, 11 months following surgery.

**Fig. 2:**
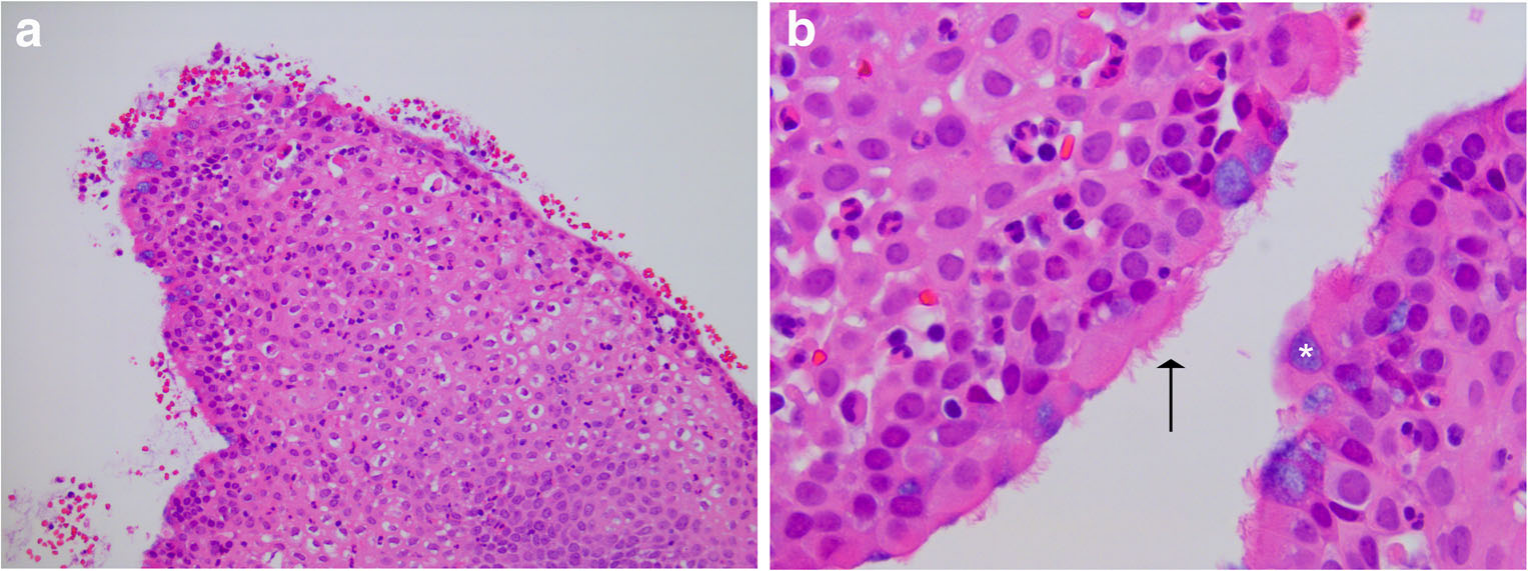
Hematoxylin and Eosin staining at × 10 magnification (**a**). Histological assessment revealed fragments of stratified squamous epithelium and an absence of wet keratin. An acute inflammatory cell infiltrate was observed throughout the tissue. High power magnification (× 40; **b**) allows appreciation of both ciliated cells (black arrow) and goblet cells (white asterisk). The underlying stroma was composed of loose connective tissue and blood vessels. Immunohistochemistry revealed an absence of intranuclear Beta-catenin staining (a feature of adamantinomatous craniopharyngiomas)

Histopathological analysis of the lesion demonstrated stratified squamous epithelium accompanied by superficial goblet cells and ciliated cells. Underlying tissue stroma comprised loose connective tissue and blood vessels. No wet keratin was identified. The epithelial cells demonstrated physiological, membranous beta-catenin staining, thereby lacking evidence for Wnt pathway activation. Suprabasal epithelial cells stained positive for CK7 and superficial epithelial cells demonstrated strong CAM5.2 positivity. CK20 staining was negative. The morphological appearances were therefore consistent with a diagnosis of papillary craniopharyngioma (Fig. 2a, b). Biological re-affirmation was sought using next-generation sequencing analysis of the lesion, which confirmed a BRAF V600E mutation (BRAFc.1799T>A p. (Val600Glu)) thereby validating the histopathological diagnosis.

## Discussion

To our knowledge, this is the youngest patient published to date with a papillary craniopharyngioma and confirmed BRAF V600E mutation.

The main differential diagnoses included adamantinomatous craniopharyngioma, Langerhans cell histiocytosis (LCH), and Rathke’s cleft cyst (RCC) with squamous metaplasia. Histological and clinical assessment discounted LCH, while the absence of Wnt pathway activation excluded an adamantinomatous craniopharyngioma. Likewise, despite sharing histological similarities to a papillary craniopharyngioma, RCC was excluded by the absence of a BRAF point mutation [15, 19, 27]. Whether a RCC can acquire a BRAF mutation and transform into a papillary craniopharyngioma remains hypothetical [26].

Papillary craniopharyngiomas are extremely rare in children. This is highlighted by a literature review of published pediatric cases (aged below 16 years), using OVID and Pubmed search with clearly defined terms (Table 1). None of the published cases identified had complete clinical information, such that the effectiveness of conventional management strategies for this patient group could not be ascertained.

**Table 1:** Defined cases of pediatric papillary craniopharyngioma published in the literature

Study (year)	Number of cases	Patient age	Gender	Hypothalamic involvement	Radiological appearance	Molecular confirmation	Therapy	Outcome
Present case (2018)	1	4 years	Female	Yes	Cystic/solid	BRAF V600E	Surgery only	Alive (11 months FU)
Schlaffer et al. (2018) [26]	1	6 years	NR	NR	NR	BRAF V600E	NR	NR
Tariq et al. (2017) [28]	2 termed pediatric (1 no detail recorded)	3 years	Male	NR	NR	Not performed	Surgery only	Alive with recurrence (2 years FU)
Cheng et al. (2016) [7]	16 termed pediatric (all no details recorded)	NR	NR	NR	NR	NR	Surgery only	15 alive, 1 dead (mean 4.5 years FU)
Brastianos et al. (2014) [6]	1	9 years	Female	NR	NR	NR	NR	NR
Zhang et al. (2002) [30]	2	NR	NR	NR	NR	NR	NR	NR
Crotty et al. (1995) [8]	1	10 years	NR	NR	NR	NR	NR	NR

*FU* follow-up, *NR* not recorded

Our patient remains stable almost 1 year following tumor surgery. Nevertheless, in adults, papillary craniopharyngiomas are often refractory to initial treatment. Therefore, the case encourages discussion on the most appropriate adjuvant therapy for young children with papillary craniopharyngiomas who have significant post-operative tumor residuum or progression. While stereotactic radiotherapy remains the recognized standard, concerns persist regarding neurocognitive, vascular, metabolic, and endocrinological sequelae, even with state-of-the-art proton beam radiotherapy [14, 20, 21].

Moreover, since most papillary craniopharyngiomas harbor the BRAF V600E oncogenic mutation [6], a novel alternative is drug inhibition of the encompassing mitogen-activated protein kinase (MAPK) pathway controlling cell division, differentiation, and invasion. BRAF inhibitors, including vemurafenib and dabrafenib, have proved successful either alone or in conjunction with trametinib, an inhibitor of another MAPK member MEK, to treat adults with papillary craniopharyngioma, resulting in tumor shrinkage with good tolerability [2, 5, 24, 25]. In addition, increasing pediatric data is emerging for BRAF and MEK inhibitors in the treatment of surgically inaccessible gliomas that express BRAF V600E mutations, with an acceptable side effect profile in this age group [1, 3, 4, 16, 18, 29]. While initial results are promising, long-term safety data is lacking as is knowledge on the longevity of therapy required.

## Conclusion

This case represents the youngest patient reported to date with a papillary craniopharyngioma that has been validated by molecular analysis, revealing a BRAF V600E mutation. While a rare occurrence, such a molecular test, is advocated if, following histopathological analysis, papillary craniopharyngioma and Rathke’s cleft cyst remain possible diagnoses. Given the burgeoning data on the efficacy and tolerability of MAPK pathway drug inhibition, this adjuvant therapeutic option warrants consideration against conventional stereotactic radiotherapy for recurrent or refractory residual pediatric papillary craniopharyngiomas.
